# Metal Work

**Published:** 1887-03

**Authors:** L. P. Haskell

**Affiliations:** Chicago


### METAL WORK.

READ BEFORE THE CENTRAL DENTAL ASSOCIATION OF NORTHERN NEW JERSEY.

BY DR. L. P. HASKELL, CHICAGO.

As everything depends upon the foundation being correct, when a metal plate is to be made be sure the impression is right. I do not deny that good impressions can in some cases be taken with wax, and more in modeling compound, but there are cases where neither is so reliable as plaster of paris. It may be accepted as an axiom, that the more difficult the case to secure an impression the greater the necessity for the use of plaster. If one uses other materials, he must select the cases most favorable for each. I have found in a long practice that plaster being always reliable, and there being no tangible objection to its use, I secure the best results when I confine myself to it.

Believing that all that is necessary to secure successful results is to have the plate come in close contact with the membrane for adhesion, with a uniform pressure on the gums in mastication and correct articulation of the teeth, the only change I make in the plaster cast is to raise it with a thin film of wax over the hard places, and scrape slightly the rear, between the center and corner of the plate. In order to have the cast deliver itself from the mould, I make the sides flaring all around, that I may secure the best results. I use the only metal which has all the requisites for a dental die—non-shrinkage, hardness, toughness, smoothness, with a melting point of low temperature. These are found in Babbitt Metal, made from the proper formula, viz: one part copper, two antimony, eight tin, care being used to melt in the order named, so that the tin shall not oxidize. The counter die should be lead, with one-sixth of tin added, so as to reduce its melting temperature, lead melting at a higher temperature than the Babbitt Metal. Coat the die with whiting before pouring the lead. It is seldom necessary to make a second die.

The principal point to guard against is the center, at the rear; see that, by pumping the moisture from under it, it sets snugly enough to exclude air, but not bearing sufficiently hard to irritate and throw off the plate.

The margins of the plate should be carried as high as they can be worn all around, especially over the cuspids. The plate will hold better, and it must be carried high over the cuspids in order to restore the lost expression resulting from the extraction of those teeth; but it must be lowered just back of them to allow full play to the muscles.

The use of gum sections is inadmissible in my practice, because I cannot secure proper arrangement of the teeth nor of the gum, so as to restore the condition of the lips; neither can proper articulation of the teeth be secured with them. Using plain teeth, then, we must use a rubber gum for attachment to the plate. I solder platinum loops to the plate to hold the rubber, fastening one end only.

As much depends upon a correct antagonism or occlusion of the teeth as upon the fit of the plate, and many cases are failures from a faulty antagonism when everything else is right.

The six anterior teeth should never meet. The pressure should be upon the bicuspids and first molar, and always be equal upon both sides. If there is a lower wisdom tooth (the first and second molars missing), it usually inclines forward, and should be avoided entirely, because if it meets the upper tooth at all it crowds the plate forward, constantly increasing the pressure as the jaws come closer together. If there remain upon the lower jaw the six anterior teeth and one or two bicuspids upon one side, and no teeth upon the other, the sooner they are removed the better for the interest of the patient, for the reason that the closure is one-sided and the plate is constantly displaced. The insertion of a partial lower set would not help matters, because very soon the artificial teeth would yield to pressure and be too short, and the uppers would meet only the natural bicuspids remaining.

The position taken by some dentists that the natural teeth should be allowed to remain under all circumstances, is a mistaken policy, resulting in great discomfort to the patient in many cases.

